# Supplementary material for: FastqPuri: high-performance preprocessing of RNA-seq data
Source: BMC Bioinformatics. 2019 May 3;20:226. doi: 10.1186/s12859-019-2799-0 (PMC6500068; doi:10.1186/s12859-019-2799-0)
Supplement: Supplementary file 2 — Archive of FastqPuri. Archive containing all files needed to install and run FastqPuri v1.0.6. Date stamp March 22, 2019. (GZ 47,819 kb) [file 12859_2019_2799_MOESM2_ESM.gz › FastqPuri-1.0.6/html/makeTree_8c.html]

FastqPuri: src/makeTree.c File Reference


FastqPuri


- src

Functions |
Variables

makeTree.c File Reference

makeTree main function
More...

`#include <stdlib.h>`  
`#include <stdio.h>`  
`#include <time.h>`  
`#include "defines.h"`  
`#include "fa_read.h"`  
`#include "tree.h"`  
`#include "init_makeTree.h"`

Include dependency graph for makeTree.c:

|  |  |
| --- | --- |
| Functions | |
| int | main (int argc, char \*argv[]) |
|  | makeTree main function |
|  | |

|  |  |
| --- | --- |
| Variables | |
| uint64\_t | alloc\_mem = 0 |
|  | |
| Iparam\_makeTree | par\_MT |
|  | |

## Detailed Description

makeTree main function

Author
:   Paula Perez paula.nosp@m.pere.nosp@m.zrubi.nosp@m.o@gm.nosp@m.ail.c.nosp@m.om

Date
:   23.08.2017 This file contains the makeTree main function. It reads a fasta file, constructs a 4-tree of depth L and stores it compressed in a file. See README\_makeTree.md for more details.

## Variable Documentation

## ◆ alloc\_mem

uint64\_t alloc\_mem = 0

global variable. Memory allocated in the heap.

## ◆ par\_MT

Iparam\_makeTree par\_MT

global variable: Input parameters of makeTree.


---

Generated on Mon Mar 19 2018 23:42:01 for FastqPuri by  

 1.8.14
